# Involvement of Histone Lysine Methylation in p21 Gene Expression in Rat Kidney* In Vivo* and Rat Mesangial Cells* In Vitro* under Diabetic Conditions

**DOI:** 10.1155/2016/3853242

**Published:** 2016-08-29

**Authors:** Xiangjun Li, Chaoyuan Li, Xiaoxia Li, Peihe Cui, Qifeng Li, Qiaoyan Guo, Hongbo Han, Shujun Liu, Guangdong Sun

**Affiliations:** ^1^Department of Experimental Pharmacology and Toxicology, School of Pharmaceutical Science, Jilin University, Changchun, Jilin 130021, China; ^2^Department of Nephrology, 2nd Hospital of Jilin University, Changchun, Jilin 130041, China; ^3^Department of Endocrinology, 208th Hospital of Chinese PLA, Changchun, Jilin 130062, China

## Abstract

Diabetic nephropathy (DN), a common complication associated with type 1 and type 2 diabetes mellitus (DM), characterized by glomerular mesangial expansion, inflammation, accumulation of extracellular matrix (ECM) protein, and hypertrophy, is the major cause of end-stage renal disease (ESRD). Increasing evidence suggested that p21-dependent glomerular and mesangial cell (MC) hypertrophy play key roles in the pathogenesis of DN. Recently, posttranscriptional modifications (PTMs) have uncovered novel molecular mechanisms involved in DN. However, precise regulatory mechanism of histone lysine methylation (HKme) mediating p21 related hypertrophy associated with DN is not clear. We evaluated the roles of HKme and histone methyltransferase (HMT) SET7/9 in p21 gene expression in glomeruli of diabetic rats and in high glucose- (HG-) treated rat mesangial cells (RMCs). p21 gene expression was upregulated in diabetic rats glomeruli; chromatin immunoprecipitation (ChIP) assays showed decreased histone H3-lysine9-dimethylation (H3K9me2) accompanied with enhanced histone H3-lysine4-methylation (H3K4me1/3) and SET7/9 occupancies at the p21 promoter. HG-treated RMCs exhibited increased p21 mRNA, H3K4me level, SET7/9 recruitment, and inverse H3K9me, which were reversed by TGF-*β*1 antibody. These data uncovered key roles of H3Kme and SET7/9 responsible for p21 gene expression* in vivo* and* in vitro* under diabetic conditions and confirmed preventive effect of TGF-*β*1 antibody on DN.

## 1. Introduction

Diabetic nephropathy (DN) is one of the most serious microvascular complications of DM and the leading cause of ESRD worldwide [1]. Hyperglycemia- and growth factors-induced glomerular and tubular hypertrophy, mesangial expansion, accumulations of extracellular matrix (ECM) in the glomerular mesangium and tubulointerstitium, basement membrane thickening, glomerular sclerosis, hyaline arteriolosclerosis, and renal dysfunction are the main characteristics of DN [2, 3]. Increased kidney size in DN is largely due to glomerular cell hypertrophy [4]. Hypertrophy of mesangial cells (MCs) is associated with the evolution of diabetic glomerulopathy [5, 6], and HG-induced renal proximal tubular cell hypertrophy was reported to be involved in DN [7]. Aberrant expression of cell cycle gene p21 plays an important role in the glomerular and tubular hypertrophy associated with DN [7–10]. To explore the precise mechanism of p21 in DN is important for developing novel strategies to suppress the development and progression of DN.

Prior studies have suggested that dysregulation of the p21 gene plays a critical role in the pathogenesis of DN, characterized by glomerular cell hypertrophy.* In vivo* studies showed that p21 null mice did not develop glomerular hypertrophy [11, 12], which strongly supported the importance of p21 in DN. In addition, several investigations have demonstrated the essential role of p21 in the senescent arrest, the molecular signature of hypertrophic changes in the early stages of DN [13–17]. Diverse factors have been reported to induce aberrant expression of p21 in MCs and experimental DN, including HG [9, 18], insulin-like-growth-factor-1 (IGF-1) [9, 19], transforming growth factor-*β*1 (TGF-*β*1) [6, 10], connective tissue growth factor (CTGF) [20], advanced glycation end products (AGEs), and their receptors (RAGE) [6]. A lot of molecular signaling pathways were involved in the induction of p21, including AMPK signaling pathways [17] and mTOR signaling [17], the HP (hexosamine pathway) [21, 22], extracellular regulated kinases (ERK1/2), p38 kinase, and c-Jun N-terminal kinase (JNK) signaling pathways [23]. However, the role of epigenetic mechanisms, especially histone lysine methylation, involved in the regulation of p21 gene transcription in DN is still unknown.

Epigenetic mechanisms have been found to play an important role in changing gene expression patterns, including posttranslational modifications (PTMs) of nucleosomal histones, DNA methylation, and miRNA regulation. PTMs such as histone lysine acetylation (HKAc) and histone lysine methylation (HKme) play key roles in the gene transcription. In general, histone H3 acetylation at lysine 9, lysine 14, lysine 18, and lysine 23, methylation of H3 lysine 4 (H3K4me), and trimethylations of H3 lysine 36 and lysine 79 (H3K36me3/H3K79me3) activate chromatin for transcription factors, while histone H3 di- or trimethylation at lysine 9 and lysine 27 silence chromatin by inhibiting transcription factors accessibility [24, 25]. Chemical modifications of the H3K4 and H3K9 have been implicated in diabetes and its complications such as DN. Several studies have found that H3K9/14Ac and H3Kme were involved in TGF-*β*1 mediated p21 mRNA expression in RMCs [10, 25] and H3K4me1 associated HMT SET7/9 played a key role in p21 gene expression. In addition, a previous study has shown that HG-induced ECM associated genes upregulation was relevant to specific histone H3 lysine methylation (H3Kme) alterations at their promoters; TGF-*β*1-specific antibody pretreatment could reverse HG-induced H3Kme levels and SET7/9 recruitment [26]. However, there is no report on the alterations of active and repressive H3Kme and key HMT SET7/9 occupancy in the glomeruli of type 1 diabetic rats as well as HG-treated RMCs, and their roles in the p21 gene expression are not clear.

We now show the decreased levels of H3K9me2, enriched H3K4me, and SET7/9 recruitment at p21 promoter in the glomeruli of STZ-induced type 1 diabetic rats associated with p21 gene upregulation; HG-induced changes of active and repressive H3Kme, SET7/9 recruitment to p21 promoter in RMCs, and TGF-*β*1-specific antibody could reverse these HG-induced specific alterations. These data demonstrate a novel epigenetic mechanism of p21 gene expression leading to hypertrophy, the main feature of DN both* in vivo* and* in vitro*.

## 2. Materials and Methods

### 2.1. Animals

Male Wistar rats (220 ± 20 g body weight) were obtained from the Experimental Animal Center of Jilin University. They were housed at 22°C with a 12/12 h light-dark cycle in a pathogen-free facility; water and rat standard diet were used to feed these rats* ad libitum*. Protocols applied for all animal studies were approved by the Ethics Committee on the Care and Use of Laboratory Animals of the Second Hospital of Jilin University (Changchun, China).

### 2.2. Induction of Diabetes

The blood glucose of all the rats was normal before the study. After being fed adaptively for 3 days, all the rats were divided into control group and streptozotocin (STZ) group; the type 1 diabetes model was fasted for 12 h and then received a single intraperitoneal (*i.p.*) injection of STZ (55 mg/kg in newly prepared 0.1 mM citrate buffer, PH 4.5; Sigma Aldrich, USA), while the control animals received the same dosage of citrate buffer. The blood glucose level of rat tails was detected using One-Touch strip on day 7 after STZ injection, and rats with blood glucose concentrations above 16.7 mmol/L were considered as diabetic models (STZ group). STZ and control rats had free access to the standard dried chow diet and water for 8 weeks; at the end of the study, all rats were sacrificed and kidneys were collected for subsequent experiments.

### 2.3. Glomeruli Isolation

Renal glomeruli were isolated from the removed kidneys of sacrificed rats by differential sieving [27], then collected, and stored at −80°C for related analysis.

### 2.4. Cell Cultures

Primary cultures of rat mesangial cells (RMCs) were performed with Wistar male rats (160–180 g) using a method published previously [26], according to the protocols approved by the Ethics Committee on the Care and Use of Laboratory Animals of the Second Hospital of Jilin University (Changchun, China). RMCs were incubated with RPMI 1640 medium that contained 5.5 mmol/L D-glucose. The following experiments were performed with 6–10 passages of RMCs. Prior to stimulation, RMCs were serum-depleted in 0.2% BSA medium for 24 h.

### 2.5. RNA Isolation and Quantitative Real-Time PCR Analysis

Total RNA was isolated from RMCs and glomeruli using TRIzol (Invitrogen, Carlsbad, CA) according to the manufacturer's instructions. 2 *μ*g isolated RNA was used in reverse transcription using TaqMan Reverse Transcription Kit (Applied Biosystems Inc., Foster City, CA, USA) according to the manufacturer's protocol. The synthesized cDNA (3 *μ*L) was used in the quantitative real-time PCR (qPCR) amplification. The specific primers were synthesized from Invitrogen (Shanghai, China) as described previously [25]. QPCRs were performed with SYBR-green reagent (Life Technologies, UK) in triplicate in a final volume of 20 *μ*L, with ABI 7300 real-time PCR thermal cycler. Dissociation curves were run to detect nonspecific amplification in order to make sure single product was amplified in each reaction. The relative level of mRNA was calculated after normalization to internal control *β*-actin gene using 2^−ΔΔCt^ method.

### 2.6. Western Blot Analysis

Proteins were extracted from glomeruli samples and homogenized in RIPA buffer (50 mM Tris-HCl, 0.5% sodium deoxycholate, 150 mM NaCl, 1% NP-40, and 0.1% SDS) with the mixture of protease and phosphatase inhibitors. Equal amounts of protein samples (30 *μ*g) were subjected to 10% SDS-PAGE gels and transferred to PVDF membranes. After being blocked with 5% milk blocking buffer for 1 h at room temperature, membrane was incubated with primary antibody against p21 (A1483, ABclonal Inc., MA, USA; 1 : 500) overnight at 4°C and then washed in Tris-buffered saline with Tween and reacted with secondary goat anti-rabbit IgG antibody for 1 h at room temperature. Proteins were detected using chemiluminescence reagents (Bio-Rad). The blots were stripped and then reprobed with a monoclonal antibody against *β*-actin (3700, Cell Signaling, Danvers, MA, USA; 1 : 1000) as an internal control. The density analysis of the immunoreactive bands was performed using ImagJ (NIH).

### 2.7. Chromatin Immunoprecipitation (ChIP) Assays

RMCs pretreated with or without TGF-*β*1-specific antibody for 1 h and then treated with NG or HG for 48 hours as previously described [26], or glomeruli isolated from control and STZ groups, were fixed with 1% formaldehyde at 37°C for 10 min and then washed twice with cold PBS plus protease inhibitors and lysed as described previously [26]; lysates were sonicated and diluted. 2 mL of the supernatant solution was incubated with antibodies against methylated histones (H3K9me2(ab1220), H3K9me3(ab8898), H3K4me1(ab8895), H3K4me2(ab32356), and H3K4me3(ab8580); Abcam, Cambridge, MA) and SET7/9 (11209, Sino Biological Inc.) at 4°C overnight. Immune complexes were obtained with protein A agarose/salmon sperm beads and washed to remove nonspecific binding; bound proteins were eluted. DNA was extracted, and qPCR was performed with the indicated primers within p21 and control cyclophilin A (CypA) promoters as previously described [25]. All reactions were performed in triplicate in a final volume of 20 *μ*L. QPCR Data were analyzed using the 2^−ΔΔCt^ method normalized with input samples. Results were expressed as fold over control.

### 2.8. Statistical Analyses

Data were presented as mean ± SEM of multiple experiments. The difference between two groups was analyzed by Student's *t*-tests and the difference among multiple groups was analyzed by one-way ANOVA tests, using the GraphPad Prism 5.0 software. *P* < 0.05 was considered to be statistically significant.

## 3. Results

### 3.1. p21 Gene Was Upregulated, Whereas, Reciprocally, Repressive H3K9me2 Level Was Decreased at Its Promoter in the Kidneys Glomeruli of STZ-Induced Rats

**Figure 1:**
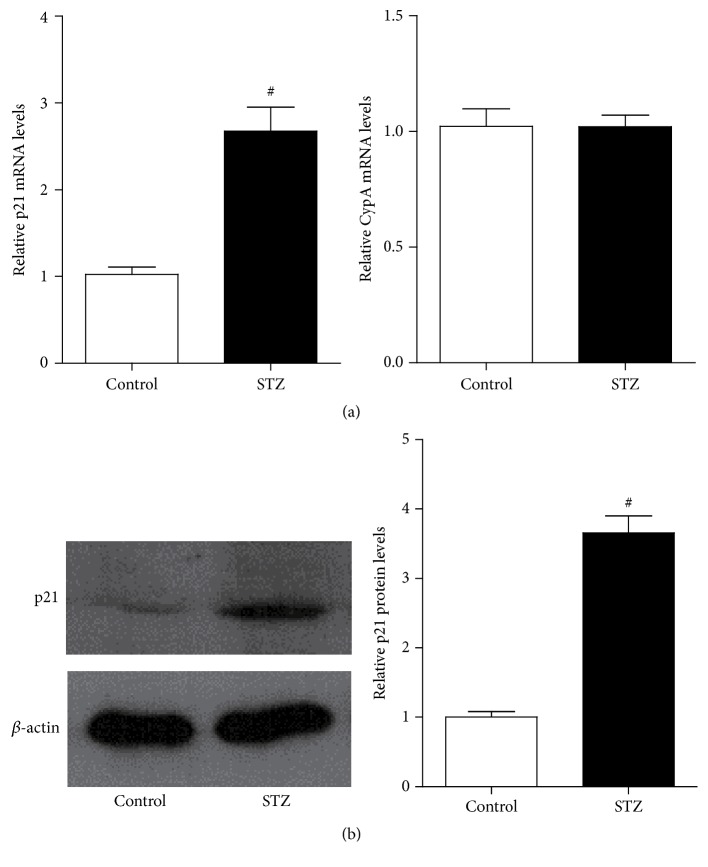
Results of p21 gene expression in the glomeruli of type 1 diabetic rats. (a) Eight weeks after male Wistar rats were successfully induced to be diabetic models with STZ (55 mg/kg), mRNA levels of p21 gene and housekeeping gene cyclophilin A (CypA) of glomeruli in control and STZ groups were analyzed by RT-qPCR. Gene expression was normalized to internal control *β*-actin gene; results were expressed as fold over control (mean ± SEM; ^#^ *P* < 0.05 compared to control, *n* = 6/group). (b) Western blot analysis of extracted proteins from control and STZ groups glomeruli using p21 and *β*-actin antibodies; quantitative analyses were expressed as fold over control (mean ± SEM; ^#^ *P* < 0.05 compared to control, *n* = 6/group).

We first observed p21 gene expression in the glomeruli of type 1 diabetic rats. Eight weeks after Wistar rats were successfully induced to be diabetic models with STZ (STZ group), glomeruli were isolated, and p21 gene expression was analyzed by RT-qPCR and western blot. p21 mRNA level increased significantly in the rat glomeruli of STZ group compared with the control group, whereas the housekeeping gene CypA showed no difference between these two groups (Figure 1(a)). In accordance with p21 mRNA expression, the protein expression of p21 was also increased in STZ group (Figure 1(b)). These results confirmed that p21 gene expression was upregulated in the glomeruli of type 1 diabetic rats.

**Figure 2:**
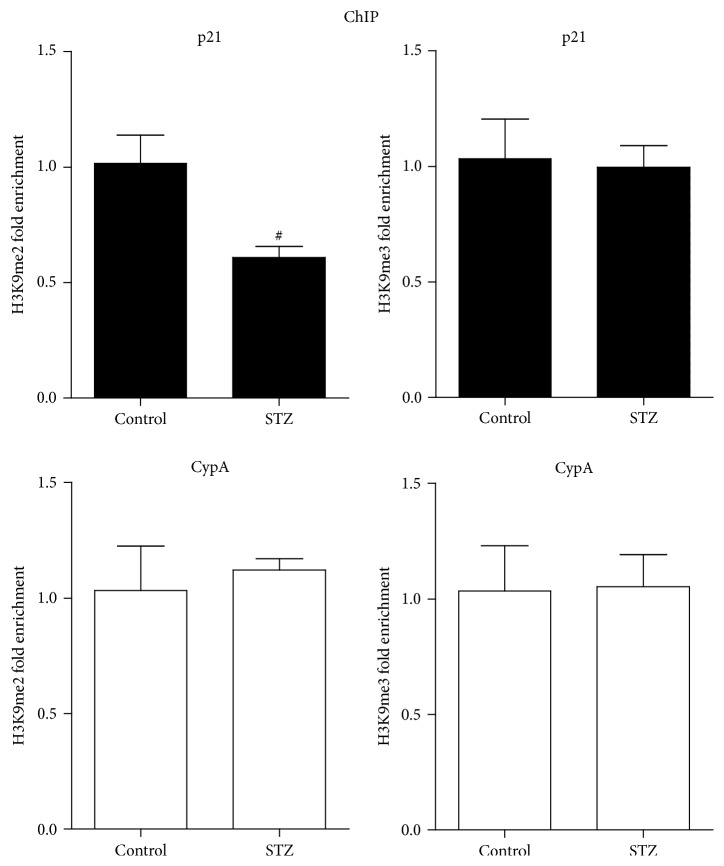
H3K9me2/3 levels at p21 gene promoter in the glomeruli of type 1 diabetic rats. Bar graphs showing H3K9me2 and H3K9me3 levels at p21 and CypA promoters in glomeruli of control and STZ groups. ChIP assays were performed with H3K9me2 and H3K9me3 antibodies, immunoprecipitated DNA and input DNA were subjected to RT-qPCR with primers for the respective promoter, data were analyzed by the 2^−ΔΔCt^ method, and results normalized to input DNA were expressed as fold over the control group (mean ± SEM; ^#^ *P* < 0.05 compared to control, *n* = 6/group).

We then examined the H3K9me2/3 levels (epigenetic “repressive” marks) at the promoter of p21 using ChIP assays with anti-histone H3 dimethyl K9 and anti-H3 trimethyl K9 antibodies. ChIP-enriched DNA samples from glomeruli were measured by quantitative PCR (qPCR) with primer at the p21 promoter as described [25]. The results indicated that H3K9me2 level (Figure 2) at the p21 promoter in the STZ group was remarkably lower compared with the control group, while there was no significant difference at the CypA promoter. The levels of H3K9me3 showed no changes between STZ and control groups. These results suggested that repressive H3K9me2 may be involved, at least in part, in the upregulation of p21 in the glomeruli of STZ-induced rats.

### 3.2. Permissive H3K4me Levels at the p21 Promoter Were Increased in the Glomeruli of Type 1 Diabetic Rats

**Figure 3:**
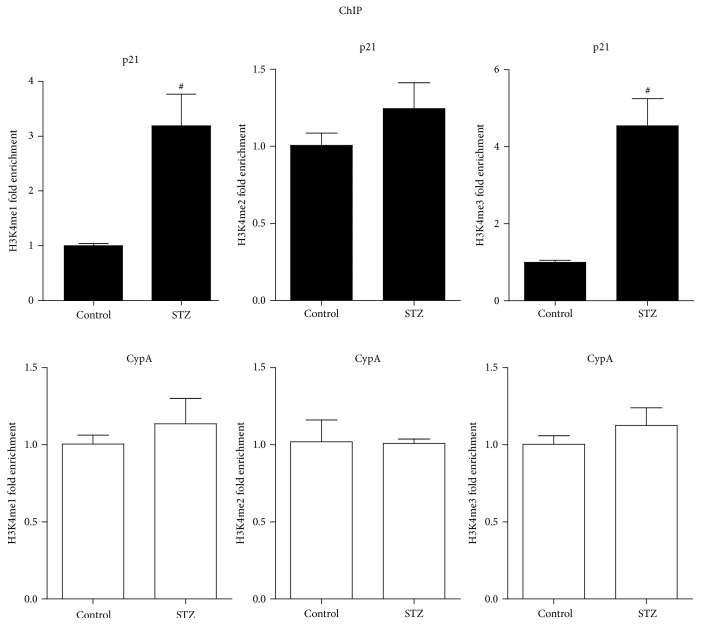
H3K4me1/2/3 levels at p21 gene promoter in the glomeruli of type 1 diabetic rats. H3K4me1, H3K4me2, and H3K4me3 levels at p21 and CypA promoters in glomeruli of control and STZ groups. ChIP assays were performed as described in Figure 2 with respective specific antibodies, and results normalized to input DNA were expressed as fold over the control group (mean ± SEM; ^#^ *P* < 0.05 compared to control).

To determine whether levels of specific activating methylation of histone H3 lysine 4 (H3K4me) were changed in the glomeruli of type 1 diabetic rats, ChIP assays were performed with H3K4me1, H3K4me2, and H3K4me3 antibodies. The results indicated that H3K4me1 and H3K4me3 levels at the p21 promoter were markedly increased in STZ group compared with control group; the levels of H3K4me2 showed no significant changes between two groups (Figure 3). These increases of H3K4me1 and H3K4me3 levels were correlative with the increased expression of p21 gene in STZ group. In contrast, the CypA promoter showed no remarkable changes in these marks, confirming specificity. These results suggested that the increases of H3K4me at the p21 promoter may be associated with the upregulation of p21 gene in the glomeruli of type 1 diabetic rats.

### 3.3. TGF-*β*1-Specific Antibody Reversed HG-Induced Reduction of Repressive H3K9me in RMCs

**Figure 4:**
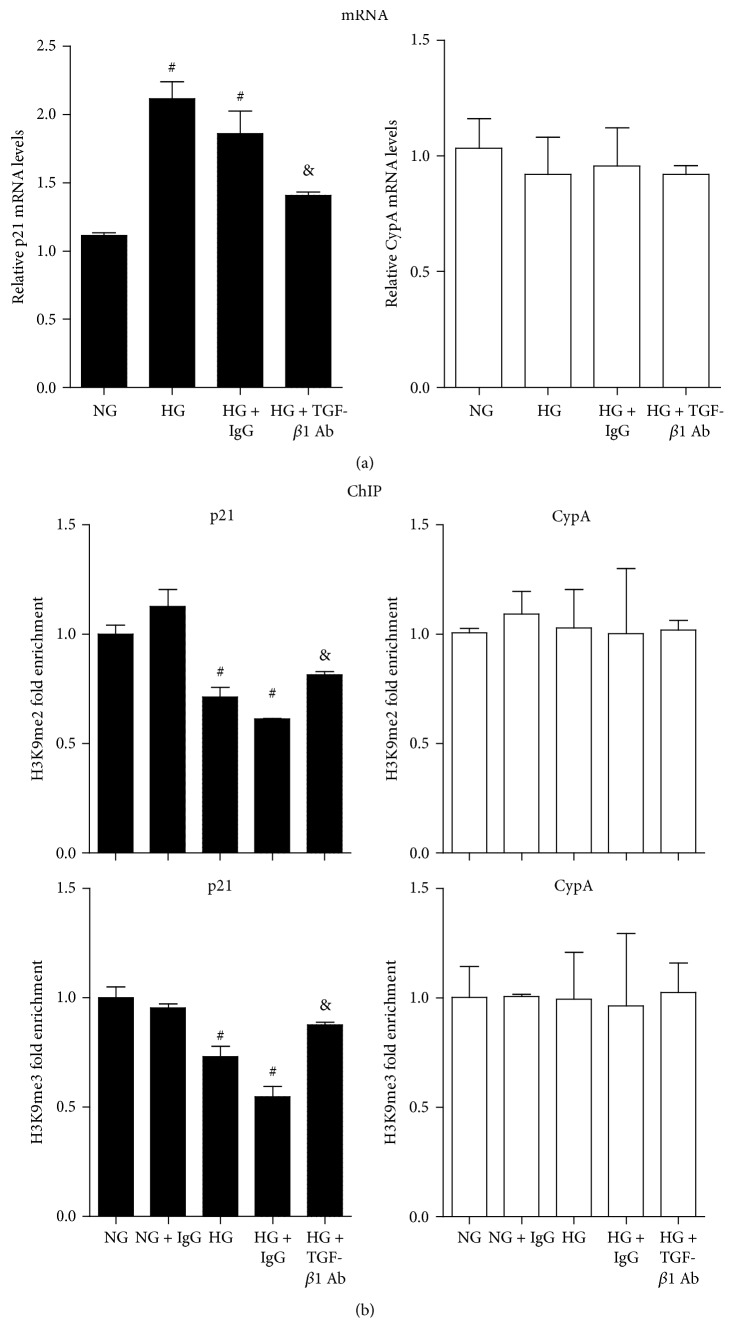
TGF-*β*1-specific antibody reversed HG-induced p21 gene expression and H3K9me2/3 changes at p21 gene promoter in RMCs. (a) p21 gene mRNA levels in RMCs. Serum-depleted RMCs were treated as described in Materials and Methods. Gene expression was analyzed by RT-qPCR, and results were expressed as fold over NG group (mean ± SEM; ^#^ *P* < 0.05 compared to NG; ^&^ *P* < 0.05 compared to HG, *n* = 3). (b) H3K9me2/3 levels at p21 and CypA gene promoters in RMCs. ChIP assays were performed as described in Materials and Methods. Results were expressed as fold over NG (mean ± SEM; ^#^ *P* < 0.05 compared to NG; ^&^ *P* < 0.05 compared to HG, *n* = 3).

We next studied HG-induced p21 gene expression and repressive H3K9me2/3 marks in RMCs. Serum-depleted RMCs were first incubated with TGF-*β*1-specific antibody or control mouse IgG (25 *μ*g/mL) for 1 h and then treated with normal glucose plus mannitol (NG, 5 mM glucose + 25 mM mannitol) or HG (30 mM glucose) for 48 hours. We first observed p21 gene expression by RT-qPCR; p21 mRNA level was significantly higher in HG group compared with NG group; the change was significantly suppressed by TGF-*β*1-specific antibody but not the control IgG, and there were no significant changes of housekeeping gene CypA among these groups (Figure 4(a)). Then we examined H3K9me levels at the promoter of p21 using ChIP-qPCRs. The results indicated that H3K9me2 and H3K9me3 levels in the HG group were markedly decreased compared with the NG group; TGF-*β*1-specific antibody rather than the IgG control pretreatment could reverse these changes, and there were no significant differences at the CypA promoter (Figure 4(b)). These results suggested that decreased repressive marks H3K9me2 and H3K9me3 may be associated with HG-induced p21 gene expression in RMCs, which could be partly reversed by TGF-*β*1-specific antibody.

### 3.4. TGF-*β*1-Specific Antibody Reversed HG-Induced Increases of H3K4me in RMCs

**Figure 5:**
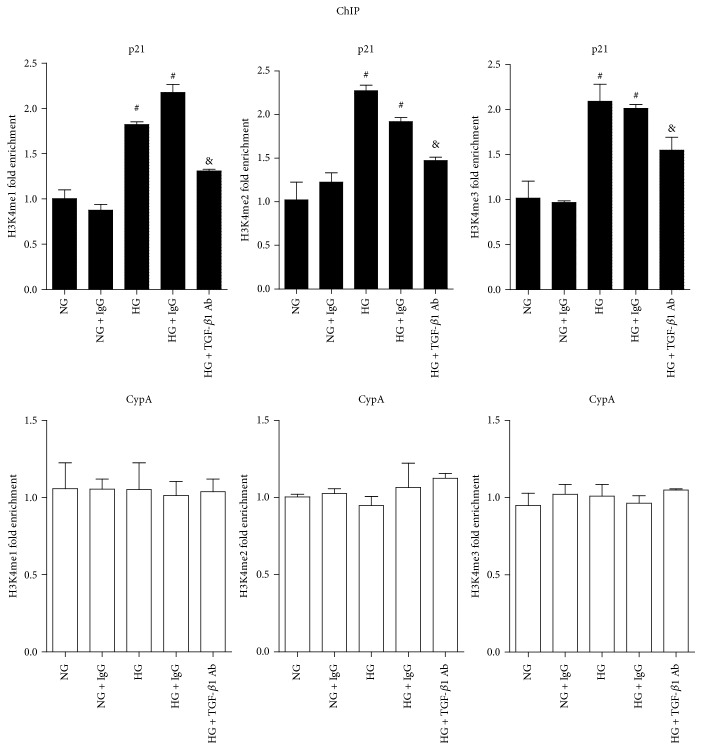
TGF-*β*1-specific antibody reversed HG-induced H3K4me1/2/3 levels at p21 gene promoter in RMCs. Bar graphs showing H3K4me1, H3K4me2, and H3K4me3 levels at p21 and CypA gene promoters in RMCs. Serum-depleted RMCs were treated as described in Materials and Methods. ChIP assays were performed as described in Materials and Methods. Results were expressed as fold over NG (mean ± SEM; ^#^ *P* < 0.05 compared to NG; ^&^ *P* < 0.05 compared to HG, *n* = 3).

We further examined whether HG could alter active H3K4me marks at the p21 promoter and whether it could be reversed by the TGF-*β*1-specific antibody. RMCs were treated as mentioned above; ChIP assays were followed with specific H3K4me1, H3K4me2, and H3K4me3 antibodies. As shown in Figure 5, H3K4me1, H3K4me2, and H3K4me3 levels at the p21 promoter were significantly increased in the HG group compared with NG group, and TGF-*β*1-specific antibody could significantly reduce the HG-induced increases. In contrast, the levels of H3K4me at the CypA promoter showed no significant differences among all the groups. These results, accompanied with the effects seen in H3K9me2/3, further reflected the mediatory role of TGF-*β*1 in HG-induced epigenetic events at the p21 promoter and its subsequent expression. Blockade of these events may be a key mechanism for the antihypertrophic and renoprotective effects of the TGF-*β*1 antibody.

### 3.5. SET7/9 Recruitment to p21 Gene Promoter Was Increased under Diabetic Conditions, and TGF-*β*1-Specific Antibody Could Reverse HG-Induced Increases in SET7/9 Recruitment in RMCs

**Figure 6:**
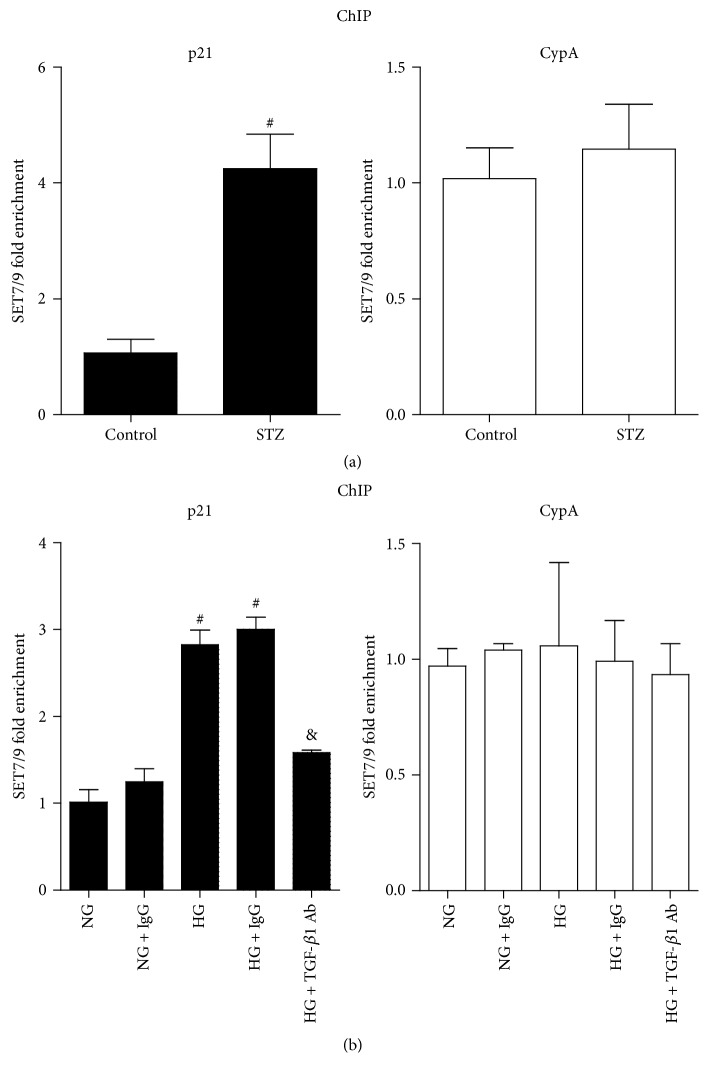
SET7/9 recruitment at p21 gene promoter was enhanced under diabetic conditions, and TGF-*β*1-specific antibody could reverse HG-induced SET7/9 occupancy in RMCs. (a) SET7/9 recruitment at p21 and CypA gene promoters in glomeruli of control and STZ groups. ChIP assays were performed and results were expressed as described in Materials and Methods (mean ± SEM; ^#^ *P* < 0.05 compared to control). (b) Reversion of HG-induced SET7/9 enrichment at p21 promoter by TGF-*β*1-specific antibody. ChIP assays were performed and results were expressed as described in Materials and Methods (mean ± SEM; ^#^ *P* < 0.05 compared to NG; ^&^ *P* < 0.05 compared to HG, *n* = 3).

To investigate the alterations of HMT SET7/9 in p21 gene expression, we first examined the SET7/9 recruitment of glomeruli in STZ-induced rats using ChIP assays with SET7/9 antibody. Results showed that SET7/9 recruitment was significantly increased at the p21 promoter in STZ group compared with control group, while there was no significant change at the CypA promoter (Figure 6(a)). The SET7/9 recruitment pattern was in accordance with the increased H3K4me1 level (Figure 3). Next, we found that HG could increase SET7/9 recruitment to the p21 promoter compared with NG in RMCs (Figure 6(b)). Moreover, TGF-*β*1-specific antibody, rather than control IgG, could significantly block the HG-induced enhancement in SET7/9 recruitment (Figure 6(b)). SET7/9 recruitment to the CypA promoter showed no differences among all of the groups. These findings indicated that there was a direct correlation between SET7/9 recruitment and increased H3K4me1 at the p21 promoter under diabetic conditions and suggested a mediatory role of TGF-*β*1 in diabetic MCs hypertrophy.

## 4. Discussion

It is widely accepted that p21 gene plays a key role in the glomerular cell hypertrophy associated with DN [8, 9, 28, 29]. p21 was reported to be upregulated by HG and other cytokines as well as growth factors, such as TGF-*β*1, CTGF, IGF-1, or AGEs [6, 8, 9, 20, 30–34], and increased in the diabetic kidney [8, 12, 35, 36]. Accumulating evidence demonstrated the key roles of histone PTMs including phosphorylation, methylation, and acetylation in the gene transcription [37, 38], and histone methylation has emerged as a prevalent modification in the gene expression. For instance, TGF-*β*1 and HG could induce significant changes of H3K9 and H3K4 methylation levels in RMCs that correlated with parallel upregulation of profibrotic genes related to ECM accumulation [26]. p21 gene expression could be modulated by PTM modifications also [39, 40]. One previous study showed that H3K9/14Ac and Smad2/3 acetylation took part in the p21 gene expression in RMCs [10]. In addition, we also found that TGF-*β*1 could change histone lysine methylation levels at the p21 promoter, which was correlated with p21 gene upregulation in RMCs [25]. However, as far as we are concerned, there is no report about histone lysine methylation and SET7/9 relative mechanism under diabetic conditions in modulating p21 gene expression.

In the current study, we confirmed that p21 gene expression was upregulated in the glomeruli of type 1 diabetic rats and could be induced by HG in RMCs; then we found significant changes of histone lysine methylation, such as decreased H3K9me2/3 and increased H3K4me1/2/3 levels and enhanced HMT SET7/9 occupancy at the p21 gene promoter, which were closely correlated with p21 gene expression both* in vivo* and* in vitro*; further results showed that TGF-*β*1-specific antibody could reverse HG-induced changes of both the histone lysine methylation and SET7/9 recruitment at p21 promoter, which correlated with the antibody-induced inhibition of p21 gene expression in RMCs.

Generally speaking, H3K9me2 and H3K9me3 have been extensively recognized as repressive histone modification associated with gene expression [25, 26]. A previous study showed that H3K9me3 levels were decreased at key inflammatory genes promoters in cultured smooth muscle cells (VSMCs) derived from db/db mice under normal and TNF-*α*-treated conditions, which were inversely correlated with upregulated expression of these inflammatory genes [41]. Furthermore, our previous ChIP results revealed that TGF-*β*1 and HG stimulation decreased both H3K9me2 and H3K9me3 levels at ECM associated genes promoters in RMCs, which were inversely associated with upregulated expression of these genes including Col1*α*1, CTGF, and PAI-1 [26]. Our recent data showed that TGF-*β*1-induced decreased H3K9me2 and H3K9me3 levels at p21 gene promoter led to increased p21 gene expression [25]. To functionally discern the role of H3K9me on the p21 gene expression, we performed ChIP assays under diabetic conditions both* in vivo* and* in vitro*. We observed a decrease of H3K9me2 level in the p21 gene promoter in the glomeruli of type 1 diabetic rats for the first time, and this inversely correlated with p21 gene upregulation; as for H3K9me3 levels, there were no differences between STZ and control groups, supporting the notion that the decrease of repressive H3K9me may partly contribute to increasing p21 gene expression* in vivo*.

Histone H3 lysine 4 methylation includes mono-, di-, and trimethylation enriched at enhancer, promoter, and other regulatory sequences, which are extensively associated with active genes expression [26, 42–44]. A previous study showed that TGF-*β*1 could induce increased levels of active chromatin marks H3K4me1/2/3 in parallel with decreased levels of repressive marks H3K9me2/3 in ECM associated genes; promoters accompanied the changes in expression of the ECM associated genes [26]; our recent study demonstrated that TGF-*β*1 could also increase H3K4me1/2/3 levels at the p21 gene promoter [25]; all these data strongly suggested the active roles for H3K4me in genes upregulation. Our results indicated that H3K4me1 and H3K4me3 levels were increased at p21 gene promoter in the glomeruli of STZ-induced diabetic rats in parallel with decreased level of H3K9me2, which was positively associated with upregulation of p21 gene expression.

Previous evidence showed that HG treatment led to the increases of H3K4me1/2/3 marks and the decreases of H3K9me2/3 levels at ECM associated genes promoters, which were correlated with HG-induced profibrotic genes expressions [26]. To further understand the key role of H3Kme in regulating HG-induced p21 expression, we investigated H3K4me1/2/3 and H3K9me2/3 levels in RMC exposed to HG; our current results showed that HG upregulated p21 gene expression, which was consistent with increased H3K4me1/2/3 and decreased H3K9me2/3 levels at the p21 gene promoter.

Several studies have demonstrated that both H3K4 HMT SET7/9 expression and SET7/9 recruitment at ECM associated genes promoters and p21 promoter were increased by TGF-*β*1 stimulation [25, 26], suggesting that SET7/9 is involved in TGF-*β*1-induced upregulations of genes associated with fibrosis and hypertrophy relevant to chronic kidney diseases including DN. To further understand the changes of HMT SET7/9 in p21 gene upregulation under diabetic conditions, we investigated the SET7/9 recruitment at p21 gene promoter in the glomeruli of STZ-induced diabetic rats and RMCs exposed to HG; the following results showed that SET7/9 occupancy was increased at p21 promoter in parallel with H3K4me1 increases both* in vivo* and* in vitro*, suggesting that SET7/9-dependent H3K4me1 plays key role in p21 gene expression leading to cellular hypertrophy associated with DN.

Accumulating evidence showed that TGF-*β*1 could lead to epigenetic changes such as H3Kme [25] and H3K9/14Ac [10] in RMCs at p21 gene promoter, which participated in p21 gene upregulation associated with MC hypertrophy in the pathogenesis of DN. TGF-*β*1-specific antibody had significant antihypertrophic effects and antifibrotic effects in both type 1 and type 2 diabetic animal models [45–47], as well as preventing HG-induced increased matrix protein synthesis in renal cells [48, 49]. A previous report showed that TGF-*β*1-specific antibody could reverse HG-induced significant changes in HKme and HMT SET7/9 recruitment at ECM associated genes promoters [26]. A recent study demonstrated that TGF-*β*1-specific antibody could significantly block the stimulatory effects of HG-induced H3K9/14Ac at the p21 promoter in RMCs [10]. Our current results provided extensive evidence that TGF-*β*1-specific antibody could reverse HG-induced significant changes in H3Kme and SET7/9 occupancy at the p21 promoter in RMCs. All the data implicated that TGF-*β*1 antibody could be an effective therapeutic agent for DN.

In summary, our data demonstrated that H3K4me and H3K9me as well as HMT SET7/9 occupancy changed significantly at p21 gene promoter in the glomeruli of type 1 diabetic rats and HG-induced RMCs, resulting in parallel increases in the p21 gene expression related to cellular hypertrophy, and that TGF-*β*1-specific antibody could reverse HG-induced changes* in vitro*, all of which are related to the pathogenesis of DN, suggesting that HKme and SET7/9 could act as potential therapeutic targets for cellular hypertrophy of DN and TGF-*β*1-specific antibody could be clinical agent for DN therapy.
